# Reference value of left and right atrial size and phasic function by SSFP CMR at 3.0 T in healthy Chinese adults

**DOI:** 10.1038/s41598-017-03377-6

**Published:** 2017-06-09

**Authors:** Weihao Li, Ke Wan, Yuchi Han, Hong Liu, Wei Cheng, Jiayu Sun, Yong Luo, Dan Yang, Yiu-Cho Chung, Yucheng Chen

**Affiliations:** 1 Cardiology Division, West China Hospital, Sichuan University, Chengdu, Sichuan Province 610041 China; 2 Radiology Department, West China Hospital, Sichuan University, Chengdu, Sichuan Province 610041 China; 3 Department of Medicine (Cardiovascular Division), University of Pennsylvania, Philadelphia, Pennsylvania USA; 4 Paul C. Lauterbur Research Centre for Biomedical Imaging, Shenzhen Key Laboratory for MRI, Shenzhen Institutes of Advanced Technology, Chinese Academy of Sciences, Shenzhen, Guangdong China

## Abstract

The size and function of the left atrium (LA) and right atrium (RA) are related closely with the prognosis of cardiovascular diseases. However, their normal reference values, as measured by cardiac magnetic resonance (CMR), are not well established in Chinese populations. Healthy Chinese subjects (n = 135, 66 males, age 23–83 years) without cardiovascular risk factors were recruited. We imaged the LA and RA of all subjects using short axis and long axis slices by steady-state free precession (SSFP) sequences using a 3.0T scanner. The size and functional parameters were measured. Age and gender differences in LA were further explored. The normal reference values of atrial dimensions, volumes, and empty fractions (EFs) were provided by short axis (SAX) and area-length methods. Volumes and EFs derived by the area-length method showed correlated well with those derived by the by SAX method, but significantly underestimated the volumes (all P < 0.001) and overestimated the LA EFs (all P < 0.001). Atrial dimensions and volumes were generally larger in males. Conduit EFs and total EFs showed gender differences. Most atrial parameters correlated with age. In general, our results showed that gender and age have considerable impact on LA and RA size and function.

## Introduction

The left atrium (LA) and right atrium (RA) are not only reservoirs, but also have active emptying functions that contribute 15–30% of ventricular filling^1, 2^. LA impairment increases with age^3, 4^, and in diseases such as hypertension^5^, heart failure^6^, atrial fibrillation^7^, hypertrophic cardiomyopathy^8^, and amyloidosis^9^. In addition, LA enlargement and LA functional changes are associated with cardiovascular mortality or worse prognosis in patients with atrial fibrillation^10, 11^, non-ischemic dilated cardiomyopathy^12^, hypertrophic cardiomyopathy^13^, and in the general population with different cardiovascular risks^14^. Compared with the LA, the RA is less studied^2, 15^, although RA function is related to the severity and prognosis of pulmonary hypertension and congenital heart disease^16, 17^.

Traditionally, two-dimensional (2D) echocardiography has been used to evaluate LA dimension and size, and the newer three-dimensional (3D) echocardiography has improved the accuracy of measurement of the atrial volume. Cardiovascular magnetic resonance imaging (CMR) has advantages in the evaluation of atrial size and phasic function compared with echocardiography and cardiac computed tomography (CT)^1, 18, 19^. CMR can provide accurate measurements of dimension, volume, and structure of the atria, with high temporal and spatial resolution. Cardiac CT also has high spatial resolution; however, radiation and nephrotoxic contrast limit its widespread use in repeated measurements. CMR is the gold standard to evaluate ventricular volumes and should also be the standard for atrial volume assessment. Accurate normal atrial reference values are crucial in clinical practice and research. Maceria *et al*. published normal atrial reference values derived from subjects of European descent^20, 21^. Similar data is not available for the Chinese population. Therefore, we aimed to provide the normal reference values for the Chinese population and study the impact of gender and age on atrial size and function.

## Materials and Methods

### Subjects

Healthy volunteers (n = 135) were recruited into this prospective study. All subjects provided a detailed history, and received a physical examination, a 12-lead electrocardiography, blood pressure measurement, and blood tests (including complete blood count), liver and renal function tests, and transthoracic echocardiography screening. The exclusion criteria were as follows: any known cardiovascular disease, hypertension, cerebrovascular disease or nervous system disease, chronic lung disease, diabetes, cancer, autoimmune diseases, recent systemic infection (within a month), recent surgery or severe trauma (within a month), any recent medications, and a history of implantation of pacemaker or other metals that are a contraindication for CMR. Subjects with abnormal findings on the comprehensive examination were also excluded. This study was approved by the ethics committee of West China Hospital, and all methods were performed in accordance with the approved guidelines. All subjects gave written informed consent.

### Cardiac Magnetic Resonance Imaging

Image acquisition was performed with a 3.0-T MRI scanner (Magnetom Tim Trio; Siemens Medical Solutions, Erlangen, Germany) using a 4-channel phased-array receiver coil combined with a spine coil. Images were acquired by steady-state free precession (SSFP) sequence during breath-holds with retrospective electrocardiogram (ECG) gating (TR, 3.4 ms; TE, 1.3 ms; flip angle, 50 degrees; FOV, 320–340 mm; matrix size, 256 × 144; and slice thickness 6 mm, with no gap). Temporal resolution was 42 ms and reconstruction in plane spatial resolution was 1.4 mm * 1.3 mm. Atrial images were acquired in consecutive short-axis views from the atria-ventricular ring to the base of the atria and in long-axis views (2-, 3-, and 4-chamber (ch)). Right ventricle (RV) 2-ch slice was performed to evaluate the RA.

### Image Analysis

#### LA Measurement

**Figure 1:**
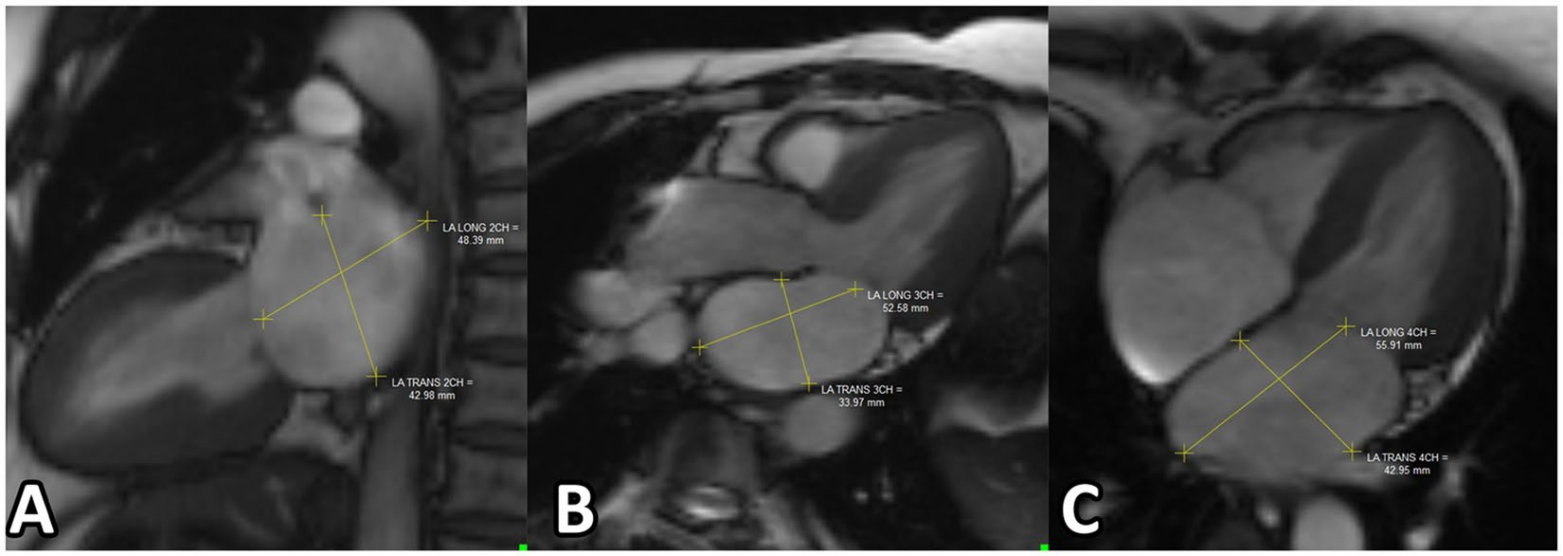
Measuring the left atrium dimension in a 2-chamber view, 3-chamber view, and 4-chamber view. (**A**) longitudinal dimension and transverse dimension in the 2-chamber view. (**B**) longitudinal dimension and antero-posterior dimension in the 3-chamber view. (**C**), longitudinal dimension and transverse dimension in the 4-chamber view. Yellow lines indicate the dimensions measured.

All CMR images were measured using a dedicated CMR post-processing software (Qmass 7.6, Medis, The Netherlands). LA dimensions were measured at the end of the systolic phase of the left ventricle (before the opening of the mitral valve) on 2-ch, 3-ch, and 4-ch SSFP cine images (Fig. 1). The LA volume was measured by two methods. First, the bi-plane area-length method with manually drawn endocardial contours in 2-and 4-ch views with exclusion of left atrial appendage and pulmonary veins^22–25^; and second, the short axis (SAX) method using Simpson’s method on the short axis slices of the atria. To calculate the atrial stoke volume and empty fraction (EF), the atrial volume at three phases during the cardiac cycle was measured. LA maximal volume (LAV_max_) was defined at the end systole before the opening of the mitral valve. LA minimal volume (LAV_min_) was defined at the end of diastole, just before the closure of the mitral valve. The pre-atrial contraction volume (LAV_p−ac_) was defined at the beginning of left atrial active contraction phase at the mid diastole of the ventricle. Parameters for atrial emptying volume and emptying function were calculated as follows:

$$\begin{array}{c}{\rm{Reservoir}}\,\mathrm{function}:\mathrm{Total}\,{\rm{EF}}( \% ):100\times ({{\rm{LAV}}}_{{\rm{\max }}}-{{\rm{LAV}}}_{{\rm{\min }}}){/\mathrm{LAV}}_{{\rm{\max }}}.\\ {\rm{Conduit}}\,\mathrm{function}:\mathrm{Conduit}\,{\rm{EF}}( \% ):100\times ({{\rm{LAV}}}_{{\rm{\max }}}-{{\rm{LAV}}}_{P-\mathrm{ac}}){/\mathrm{LAV}}_{{\rm{\max }}}.\\ {\rm{Booster}}\,\mathrm{function}:\mathrm{Booster}\,{\rm{pump}}\,{\rm{EF}}( \% ):100\times ({{\rm{LAV}}}_{p-\mathrm{ac}}-{{\rm{LAV}}}_{{\rm{\min }}}){/\mathrm{LAV}}_{p-\mathrm{ac}}\end{array}$$

The indexed dimension and volume values were calculated by the corresponding values divided by body surface area (BSA). The BSA values were derived from the height and weight by the DuBois & DuBois formula (BSA = (W^0.425^ × H^0.725^) × 0.007184)^26^.

#### RA Measurement

**Figure 2:**
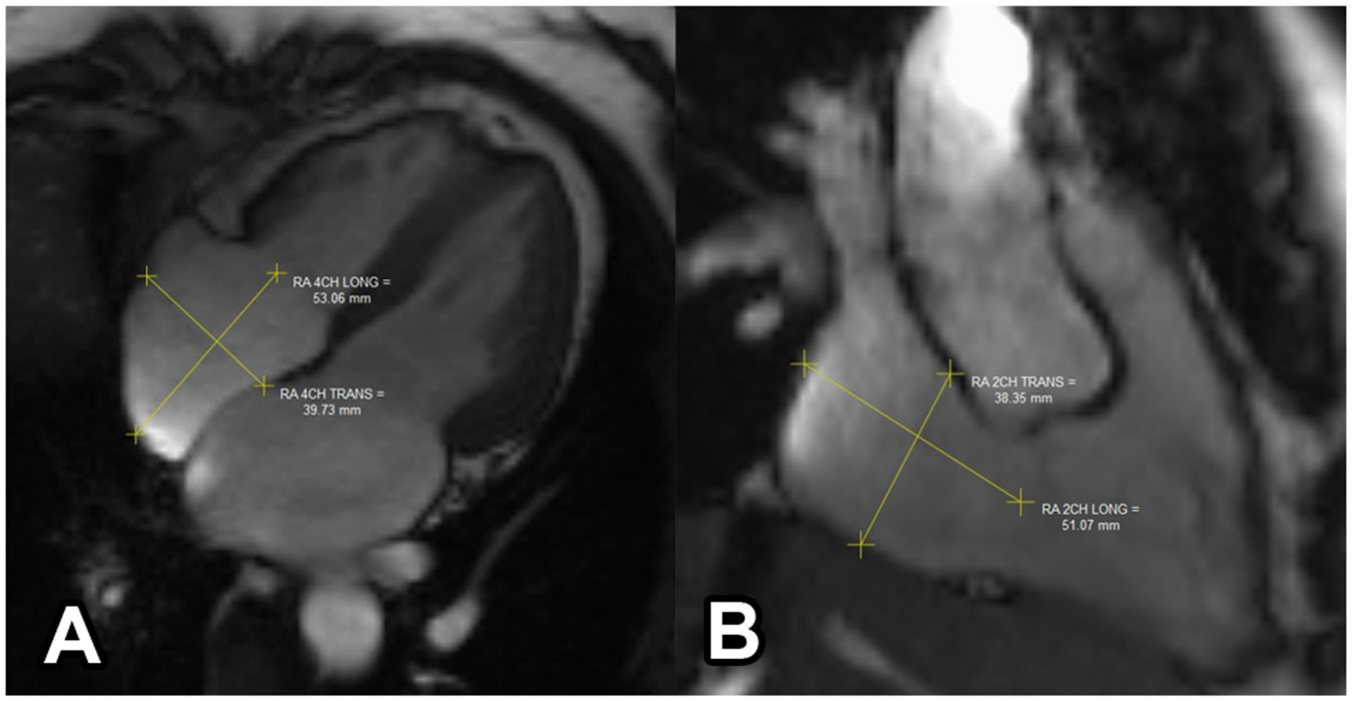
Measuring the right atrium dimensions in a 4-chamber view and a right ventricle (RV) 2-chamber view. (**A**) longitudinal dimension and transverse dimension in the right ventricle 2-chamber view. (**B**) longitudinal dimension and transverse dimension in the 4-chamber view. Yellow lines indicate the dimensions measured.

The dimensions of the RA were measured on 4-ch SSFP images and RV 2-ch SSFP images (Fig. 2). The RA volume was measured by the area-length method and the SAX method, similar to the LA. Single plane area-length and bi-plane area length were both used to calculate the RA volume^27^. Similar to the measurement of the LA, maximal RA volume (RAV_max_), minimal RA volume (RAV_min_), and pre-active contraction RA volume (RAV_p−ac_) were acquired at the same phases as the LA. RA phasic functions were defined the same as LA phasic function. RA total emptying fraction, RA passive emptying fraction, and RA active emptying fraction were calculated using the same formulas. Similarly, indexed dimension and volume values were also calculated for the RA.

### Inter-observer and Intra-observer Variability

Subjects (20%, 24 cases) were selected randomly to test inter- and intra-observer variability. For inter-observer variability, two independent observers (WHL and KW), with more than 2 years experience and 500 cases of CMR image analysis, finished the post-processing for atrial dimensions and volumes blindly. For intra-observer variability, one observer (WHL) repeated the measurements for all parameters using the identical methods 8 weeks apart.

### Statistical Analyses

Statistical analyses were performed using SPSS (version 17.0; SPSS Inc., Chicago, IL, USA) and MedCalc (MedCalc Software version 13.0; Ostend, Belgium). The Kolmogorov-Smirnov test was used to check the normal distribution of the continuous variables. Independent-sample *T* tests were used to compare the mean values between men and women. Continuous data are presented as the mean ± SD. Non-normally distributed data were converted into log (normally distributed data), and then expressed as the mean ± SD. The normal reference range was calculated as the mean ± 2 SD. Linear regression was used to analyse the relationships between cardiac parameters and age. The inter- and intra-observer variability was assessed using the Bland-Altman method. A P value of <0.05 was considered statistically significant.

### Data Availability

The datasets is available from the corresponding author on reasonable request.

## Results

### Subject Demographic Data

The demographic data of the 135 healthy volunteers are shown in the Table 1. The average age in this group was 49.9 ± 17.1 years, and 49% were males.

**Table 1:** Demographic characteristics.

Parameters	Total (mean ± SD)	Male (mean ± SD)	Female (mean ± SD)	P (Male *vs*. Female)
Subjects number	135	66	69	
Age (years)	49.9 ± 17.1	50.5 ± 17.2	49.2 ± 17.2	0.665
Age range (years)	23 to 83	23 to 77	23 to 83	
Height (cm)	160.8 ± 8.9	167.4 ± 7.0	154.4 ± 5.0	<0.001
Weight (kg)	58.5 ± 9.6	64.3 ± 8.9	53.0 ± 6.6	<0.001
BMI (kg/m^2^)	22.6 ± 2.8	22.9 ± 2.9	22.3 ± 2.7	0.162
BSA (m^2^)	1.6 ± 0.2	1.7 ± 0.1	1.5 ± 0.1	<0.001
SBP (mmHg)	118.3 ± 10.7	120.8 ± 9.9	115.9 ± 10.8	0.007
DBP (mmHg)	78.9 ± 9.2	81.7 ± 9.0	76.2 ± 8.5	<0.001
HR (bpm)	72.1 ± 8.8	71.3 ± 9.0	72.9 ± 8.5	0.284

BMI, body mass index, calculated by (weight in kg)/(height in m)^2^; BSA, body surface area, calculated by DuBois & DuBois formula; SBP, systolic blood pressure; DBP, diastolic blood pressure; HR, heart rate.

### Normal Reference Values for LA

#### LA Dimensions

The normal LA dimensions and indexed values are shown in Table 2. Most dimensions showed no gender differences. The anterior-posterior dimension on the 3-ch view was greater in females than in males (31.1 ± 5.5 *vs*. 28.7 ± 5.3 mm, P = 0.011). The longitudinal diameter on 4-ch was shorter in females than in males (55.2 ± 6.0 *vs*. 57.9 ± 5.7 mm, P = 0.008). However, after indexing by BSA, the indexed LA diameters in females were slightly greater than those in males (All P < 0.001).

**Table 2:** Total and gender specific left atrial dimensions (mean ± SD, and reference range) (n = 135).

Parameters	Total (n = 135)		Male (n = 66)		Female (n = 69)		P (Male *vs*. Female)
	Mean ± SD	Lower/upper limits	Mean ± SD	Lower/upper limits	Mean ± SD	Lower/upper limits	
Long. − 2ch[mm]	50.1 ± 6.3	(37.6, 62.6)	51.2 ± 6.2	(38.8, 63.7)	49.1 ± 6.1	(36.8, 61.3)	0.046
Trans. − 2ch [mm]	42.1 ± 5.5	(31.1, 53.2)	42.5 ± 6.5	(29.5, 55.6)	41.8 ± 4.4	(33.0, 50.5)	0.425
Long. − 3ch [mm]	54.4 ± 6.6	(41.1, 67.6)	55.1 ± 6.4	(42.3, 67.9)	53.7 ± 6.8	(40.1, 67.3)	0.239
AP − 3ch [mm]	29.9 ± 5.5	(18.9, 41.0)	28.7 ± 5.3	(18.1, 39.3)	31.1 ± 5.5	(20.1, 42.1)	0.011
Long. − 4ch [mm]	56.5 ± 6.0	(44.6, 68.5)	57.9 ± 5.7	(46.5, 69.3)	55.2 ± 6.0	(43.3, 67.1)	0.008
Trans. − 4ch [mm]	41.0 ± 4.8	(31.3, 50.7)	41.6 ± 4.9	(31.8, 51.4)	40.4 ± 4.8	(30.9, 49.9)	0.169
Indexed Long. − 2ch [mm/m^2^]	31.5 ± 4.7	(22.1, 41.0)	30.0 ± 4.2	(21.5, 38.5)	33.0 ± 4.7	(23.6, 42.4)	<0.001
Indexed Trans. − 2ch [mm/m^2^]	26.4 ± 3.7	(18.9, 33.9)	24.8 ± 3.8	(17.2, 32.4)	28.0 ± 2.9	(22.1, 33.9)	<0.001
Indexed Long. − 3ch [mm/m^2^]	34.2 ± 4.8	(24.5, 43.8)	32.2 ± 4.1	(23.9, 40.4)	36.1 ± 4.7	(26.7, 45.4)	<0.001
Indexed AP − 3ch [mm/m^2^]	18.8 ± 3.8	(11.3, 26.3)	16.7 ± 2.8	(11.0, 22.3)	20.8 ± 3.4	(14.0, 27.7)	<0.001
Indexed Long. − 4ch [mm/m^2^]	35.5 ± 4.4	(26.8, 44.2)	33.8 ± 3.9	(26.0, 41.7)	37.0 ± 4.2	(28.7, 45.4)	<0.001
Indexed Trans. − 4ch [mm/m^2^]	25.7 ± 3.5	(18.8, 32.7)	24.2 ± 2.8	(18.6, 29.9)	27.1 ± 3.5	(20.2, 34.1)	<0.001

Lower/upper limits calculated as mean ± 2 SD; Long., Longitudinal dimension; Trans, Transverse dimension; AP, Antero-posterior dimension; 2ch, 2-chamber view; 3ch, 3-chamber view; 4ch, 4-chamber view; Indexed dimensions are calculated by corresponding dimensions in mm divided by BSA in m^2^.

#### LA Volume and Phasic Function

The LA volume parameters are shown in Table 3. Correlations between parameters measured by the SAX method and the bi-plane method were moderate. Compared with the SAX method, the bi-plane method underestimated the LA volumes and overestimated the phasic function (all P < 0.001). The LA volumes in females were significantly lower than those in males, except for LAV_max_ (P = 0.119 for SAX method and 0.090 for bi-plane area-length method, all others P < 0.05, namely 0.009 for the LAV_p−ac_ SAX method, 0.008 for the LAV_min_ SAX method, 0.004 for the LAV_p−ac_ bi-plane area-length method, 0.020 for the LAV_min_ bi-plane area-length method) (Table 4). However, after indexing by BSA, most of the volume parameters were similar between genders (P = 0.668 for the indexed LAV_p−ac_ SAX method, P = 0.654 for the indexed LA_min_ SAX method, P = 0.096 for the indexed LA_max_ bi-plane area-length method, P = 0.755 for the indexed LAV_p−ac_ bi-plane area-length method, and P = 0.949 for the indexed LAV_min_ bi-plane area-length method), except for LAV_max_ by the SAX method (female *vs*. male: 43.9 ± 8.2 mL/m^2^ *vs*. 40.05 ± 8.3 mL/m^2^, P = 0.018). The LA conduit EF was greater in females than in males when measured by either the SAX method or the bi-plane method (P = 0.004 and 0.008, respectively), while there was no significant difference in booster pump EF and total LA EF (P = 0.984 for the booster pump EF by the SAX method, P = 0.372 for the booster pump EF by the bi-plane area-length method, P = 0.095 for the total EF by the bi-plane area-length method, and P = 0.654 for the total EF by the SAX method).

**Table 3:** Short axis *vs*. bi-plane area-length left atrial volume parameters in the whole group (n = 135).

Parameter	Short axis method		Bi-plane area-length method		P (bi-plane *vs*. sax)	Difference (Mean ± SE)	Pearson Correlation	P
	Mean ± SD	Lower/upper limits	Mean ± SD	Lower/upper limits				
LAV_max_, in ml	67.6 ± 14.2	(39.1, 96)	61.9 ± 14.7	(32.5, 91.4)	<0.001	−5.6 ± 1.1	0.648	0.000
LAV_p−ac_, in ml	48.9 ± 13.1	(22.7, 75.1)	41.5 ± 12.8	(15.9,67.1)	<0.001	−7.4 ± 0.9	0.676	0.000
LAV_min_, in ml	30.2 ± 8.0	(14.2, 46.1)	25.1 ± 8.9	(7.4, 42.8)	<0.001	−5.1 ± 0.7	0.594	0.000
Indexed LAV_max_, in ml/m^2^	42.2 ± 8.4	(25.4, 59.1)	38.7 ± 9.0	(20.8, 56.7)	<0.001	−3.5 ± 0.6	0.632	0.000
Indexed LAV_p−ac_, in ml/m^2^	30.5 ± 7.6	(15.4, 45.6)	25.9 ± 7.6	(10.7, 41.1)	<0.001	−4.6 ± 0.6	0.644	0.000
Indexed LAV_min_, in ml/m^2^	18.8 ± 4.5	(9.8, 27.8)	15.7 ± 5.4	(5.0, 26.4)	<0.001	−3.1 ± 0.4	0.566	0.000
Conduit EF, %	28 ± 9	(10, 46)	33 ± 10	(12, 54)	<0.001	5 ± 1	0.594	0.000
Booster pump EF, %	37 ± 10	(18, 57)	39 ± 12	(16, 62)	0.045	2 ± 1	0.563	0.000
Total EF, %	55 ± 8	(40, 70)	60 ± 8	(43, 76)	<0.001	5 ± 1	0.395	0.000

Lower/upper limits calculated as mean ± 2 SD; LAV_max_, maximal left atrial volume; LAV_p−ac_, left atrial volume prior to atrial contraction; LAV_min_, minimal left atrial volume; Indexed volumes are calculated by corresponding volume in ml divided by BSA in m^2^; Conduit EF, Conduit left atrial emptying fraction: 100% × (LAV_max_ − LAV_p−ac_)/LAV_max_; Booster pump EF, Booster pump left atrial emptying fraction: 100% × (LAV_p−ac_ − LAV_min_)/LAV_p−ac_; Total EF, total left atrial emptying fraction: 100% × (LAV_max_ − LAV_min_)/LAV_max_.

**Table 4:** Gender specific short axis and bi-plane area-length reference values of LA volume and phasic function (n = 135).

Parameter	Short axis method					bi-plane area-length method				
	Male		Female		P (Male *vs*. Female)	Male		Female		P (Male *vs*. Female)
	Mean ± SD	Lower/upper limits	Mean ± SD	Lower/upper limits		Mean ± SD	Lower/upper limits	Mean ± SD	Lower/upper limits	
LAV_max_, in ml	69.5 ± 14.9	(39.7, 99.4)	65.7 ± 13.4	(39, 92.4)	0.119	64.1 ± 15.6	(33, 95.3)	59.8 ± 13.6	(32.5, 87.1)	0.090
LAV_p−ac_, in ml	51.9 ± 13.8	(24.3, 79.5)	46.00 ± 11.8	(22.4, 69.5)	0.009	44.8 ± 14.4	(15.9, 73.6)	38.4 ± 10.2	(18.0, 58.7)	0.004
LAV_min_, in ml	32.1 ± 8.8	(14.5, 49.6)	28.4 ± 6.7	(14.9, 41.8)	0.008	26.9 ± 10.1	(6.8, 47.0)	23.4 ± 7.2	(8.9, 37.8)	0.020
Indexed LAV_max_, in ml/m^2^	40.5 ± 8.3	(23.9, 57.1)	43.9 ± 8.2	(27.4, 60.4)	0.018	37.4 ± 9.1	(19.2, 55.7)	40.0 ± 8.7	(22.6, 57.4)	0.096
Indexed LAV_p−ac_, in ml/m^2^	30.2 ± 7.7	(14.8, 45.6)	30.8 ± 7.5	(15.8, 45.7)	0.668	26.1 ± 8.5	(9.0, 43.2)	25.7 ± 6.7	(12.4, 39.0)	0.755
Indexed LAV_min_, in ml/m^2^	18.6 ± 4.8	(9.0, 28.2)	19 ± 4.2	(10.5, 27.4)	0.654	15.7 ± 6	(3.7, 27.7)	15.7 ± 4.7	(6.2, 25.1)	0.949
Conduit EF, %	26 ± 9	(8, 44)	30 ± 9	(13, 48)	0.004	31 ± 10	(11, 51)	35 ± 10	(15, 56)	0.008
Booster pump EF, %	37 ± 10	(17, 58)	37 ± 10	(18, 57)	0.984	39 ± 13	(13, 66)	39 ± 10	(19, 59)	0.372
Total EF, %	54 ± 8	(37, 70)	57 ± 6	(44, 70)	0.028	59 ± 9	(41, 76)	61 ± 8	(46, 76)	0.095

Lower/upper limits calculated as mean ± 2 SD; LAV_max_, maximal left atrial volume; LAV_p−ac_, left atrial volume prior to atrial contraction; LAV_min_, minimal left atrial volume; Indexed volumes are calculated by corresponding volume in ml divided by BSA in m^2^; Conduit EF, Conduit left atrial emptying fraction: 100% × (LAV_max_ − LAV_p−ac_)/LAV_max_; Booster pump EF, Booster pump left atrial emptying fraction: 100% × (LAV_p−ac_ − LAV_min_)/LAV_p−ac_; Total EF, total left atrial emptying fraction: 100% × (LAV_max_ − LAV_min_)/LAV_max_.

### Normal Reference for RA

#### RA Dimensions

The linear RA dimensions measured on the 4-ch view and RV 2-ch view are shown in Table 5. The absolute RA dimensions were similar in males and females (P = 0.193 for longitudinal diameter in 2-ch, P = 0.581 for longitudinal diameter in 4-ch, and P = 0.127 for transverse diameter in 2-ch, except P < 0.001 for the transverse dimension in 4-ch), while the indexed diameters were higher in females than in males (P < 0.001 for the indexed longitudinal diameter in 2ch and 4ch, P = 0.020 for the indexed transverse diameter in 4ch), except for the indexed transverse diameter in 2ch, where P = 0.087).

**Table 5:** Total and gender specific right atrial dimension parameters (mean ± SD, and reference range) (n = 135).

Parameters	Total		Male		Female		P (Male *vs*. Female)
	Mean ± SD	Lower/upper limits	Mean ± SD	Lower/upper limits	Mean ± SD	Lower/upper limits	
Long. − 2ch [mm]	51.3 ± 6.6	(38.1, 64.4)	52.1 ± 6.0	(40.0, 64.1)	50.6 ± 7.0	(36.5, 64.6)	0.193
Trans. − 2ch [mm]	39.6 ± 9.8	(19.9, 59.2)	40.9 ± 10.2	(20.5, 61.4)	38.3 ± 9.3	(19.7, 56.9)	0.127
Long. − 4ch [mm]	50.1 ± 5.7	(38.7, 61.5)	50.4 ± 5.8	(38.9, 61.9)	49.9 ± 5.7	(38.4, 61.3)	0.581
Trans. − 4ch [mm]	43.5 ± 5.4	(32.7, 54.3)	45.6 ± 4.9	(35.7, 55.4)	41.5 ± 5.1	(31.2, 51.8)	<0.001
Indexed Long. − 2ch [mm/m2]	32.3 ± 4.6	(23.0, 41.5)	30.5 ± 3.9	(22.7, 38.2)	33.9 ± 4.7	(24.5, 43.3)	<0.001
Indexed Trans. − 2ch [mm/m2]	24.9 ± 6.4	(12.1, 37.6)	23.9 ± 5.9	(12.2, 35.7)	25.8 ± 6.7	(12.4, 39.2)	0.087
Indexed Long. − 4ch [mm/m2]	31.5 ± 4.5	(22.5, 40.5)	29.5 ± 4.0	(21.4, 37.5)	33.5 ± 4.1	(25.4, 41.6)	<0.001
Indexed Trans. − 4ch [mm/m2]	27.2 ± 3.2	(20.9, 33.6)	26.6 ± 2.8	(20.9, 32.2)	27.8 ± 3.4	(21.1, 34.6)	0.020

Lower/upper limits calculated as mean ± 2 SD; Long., Longitudinal dimension; Trans, Transverse dimension; AP, Antero-posterior dimension; 2ch, 2-chamber view; 3ch, 3-chamber view; 4ch, 4-chamber view; Indexed dimensions are calculated by corresponding dimensions in mm divided by BSA in m^2^.

#### RA Volume and Phasic Function

The RA volume and phasic function data are shown in Table 6. Compared with the SAX method, the absolute volume and indexed volume measured by either single plane or bi-plane area-length methods were much lower (all P < 0.001). Correlations between the SAX and the area-length methods were moderate. The phasic functions of the RA were similar when assessed by the two methods (all P > 0.05, namely P = 0.278 for the conduit EF by the bi-plane area-length method *vs*. the SAX method, P = 0.209 for the total EF by the bi-plane area-length method *vs*. the SAX method, P = 0.064 for the conduit EF area-length in the 4ch method *vs*. the SAX method, P = 0.073 for the booster pump EF area-length in the 4ch method *vs*. the SAX method, P = 0.369 for the total EF area-length in the 4ch method vs. the SAX method, except P = 0.002 for the booster pump EF by the bi-plane area-length method *vs*. the SAX method). The absolute RA volume was larger in males than in females (All P < 0.001), and this difference persisted for a number of methods after indexing by BSA (P = 0.021 for indexed RAV_p−ac_ by the SAX method, P = 0.001 for indexed RAV_p−ac_ by the bi-plane area-length method, P = 0.005 for indexed RAV_min_ by the SAX method, P = 0.001 for indexed RAV_min_ by the bi-plane area-length method, P = 0.011 for indexed RAV_min_ area-length in the 4ch method, except for 0.070 for indexed RAV_p−ac_ area-length in the 4ch method), except for the RV maximal volume index (P = 0.678 for indexed RAV_max_ by the SAX method, P = 0.181 for indexed RAV_max_ by the bi-plane area-length method, and P = 0.142 for indexed RAV_max_ by the SAX method) (Table 7). RA conduit EF and RA total EF were higher in females than in males by either method (for conduit EF, P = 0.003 by the SAX method, P < 0.001 by the bi-plane area-length method, and P < 0.001 by the area-length method in the 4-ch view; for total EF, P < 0.001 by the SAX method, P = 0.001 by the bi-plane area-length method, and P = 0.010 by the area-length method in the 4-ch view). The RA booster pump EF showed no gender difference (P = 0.092 by SAX, P = 0.152 by bi-plane area-length, and P = 0.660 by the area-length method in the 4-ch view).

**Table 6:** Right atrial volume and phasic function parameters measured by the short axis or area-length method (n = 135).

Parameter	Short axis method		Bi-plane area-length method						Area-length method (4-chamber)					
	Mean ± SD	Lower/upper limits	Mean ± SD	Lower/upper limits	P (sax vs. bi-plane)	Difference (Mean ± SE)	Pearson’s Correlation	P	Mean ± SD	Lower/upper limits	P (sax vs. 4ch)	Difference (Mean ± SE)	Pearson’s Correlation	P
RAV_max_, in ml	82.7 ± 19.8	(43.2, 122.3)	58.3 ± 18.5	(21.4, 95.2)	<0.001	24.1 ± 1	0.825	<0.001	59.6 ± 18.3	(22.9, 96.2)	<0.001	23.4 ± 1.3	0.699	<0.001
RAV_p−ac_, in ml	61.5 ± 16.8	(27.8, 95.2)	43.8 ± 14.9	(14, 73.6)	<0.001	17.4 ± 0.9	0.786	<0.001	45.6 ± 16.2	(13.2, 78)	<0.001	16 ± 1.1	0.704	<0.001
RAV_min_, in ml	40.3 ± 14.3	(11.6, 69)	27.5 ± 10.5	(6.4, 48.6)	<0.001	12.5 ± 0.8	0.737	<0.001	28.9 ± 12.7	(3.5, 54.3)	<0.001	11.4 ± 1	0.636	<0.001
Indexed RAV_max_, in ml/m2	51.6 ± 11.0	(29.6, 73.6)	36.3 ± 10.6	(15.2, 57.4)	<0.001	15.1 ± 0.6	0.797	<0.001	37 ± 10.3	(16.3, 57.6)	<0.001	14.7 ± 0.8	0.631	<0.001
Indexed RAV_p−ac_, in ml/m2	38.2 ± 9.0	(20.2, 56.2)	27.2 ± 8.4	(10.5, 43.9)	<0.001	10.8 ± 0.5	0.740	<0.001	28.2 ± 9.2	(9.9, 46.5)	<0.001	10 ± 0.7	0.631	<0.001
Indexed RAV_min_, in ml/m2	24.9 ± 7.7	(9.5, 40.4)	17.0 ± 5.9	(5.2, 28.9)	<0.001	7.7 ± 0.5	0.691	<0.001	17.9 ± 7.3	(3.3, 32.5)	<0.001	7 ± 0.6	0.567	<0.001
Conduit EF, %	25 ± 10	(4, 46)	25 ± 10	(4, 45)	0.278	0 ± 0	0.539	<0.001	24 ± 10	(3, 44)	0.064	0 ± 0	0.557	<0.001
Booster pump EF, %	35 ± 11	(13, 56)	38 ± 8	(21, 55)	0.002	0 ± 0	0.295	0.001	37 ± 11	(15, 59)	0.073	0 ± 0	0.299	0.001
Total EF, %	52 ± 10	(32, 71)	53 ± 9	(35, 72)	0.209	0 ± 0	0.373	<0.001	52 ± 11	(30, 74)	0.761	0 ± 0	0.369	<0.001

Lower/upper limits calculated as mean ± 2 SD; RAV_max_, maximal right atrial volume; RAV_p−ac_, right atrial volume prior to atrial contraction; RAV_min_, minimal right atrial volume; Indexed volumes are calculated by corresponding volume in ml divided by BSA in m^2^; Conduit EF, Conduit right atrial emptying fraction: 100% × (RAV_max_ − RAV_p−ac_)/RAV_max_; Booster pump EF, Booster pump right atrial emptying fraction: 100% × (RAV_p−ac_ − RAV_min_)/RAV_p−ac_; Total EF, total right atrial emptying fraction: 100% × (RAV _max_ − RAV_min_)/RAV_max_.

**Table 7:** Gender specific reference values of the RA volume and phasic function assessed by the short axis or area–length method (n = 135).

Parameter	Short axis method					Bi-plane area-length method					Area-length method (4-chamber view)				
	Male		Female		P (Male *vs*. Female)	Male		Female		P (Male *vs*. Female)	Male (n = 66)		Female (n = 69)		P (Male *vs*. Female)
	Mean ± SD	Lower/upper limits	Mean ± SD	Lower/upper limits		Mean ± SD	Lower/upper limits	Mean ± SD	Lower/upper limits		Mean ± SD	Lower/upper limits	Mean ± SD	Lower/upper limits	
RAV _max_, in ml	89.4 ± 21.5	(46.5, 132.3)	76.5 ± 15.8	(44.9, 108.1)	<0.001	64.6 ± 20.4	(23.8, 105.3)	52.5 ± 14.4	(23.8, 81.2)	<0.001	65.9 ± 19.1	(27.6, 104.1)	53.5 ± 15.4	(22.7, 84.4)	<0.001
RAV_p−ac_, in ml	69.0 ± 18.8	(31.5, 106.6)	54.4 ± 10.9	(32.5, 76.3)	<0.001	50.8 ± 15.9	(18.9, 82.6)	37.3 ± 10.3	(16.6, 57.9)	<0.001	52.3 ± 16.4	(19.4, 85.1)	39.2 ± 13.2	(12.8, 65.7)	<0.001
RAV_min_, in ml	46.4 ± 16.3	(13.7, 79.1)	34.5 ± 9.0	(16.4, 52.6)	<0.001	32.1 ± 11.6	(9, 55.3)	23 ± 7	(9, 37)	<0.001	33.6 ± 14.2	(5.2, 61.9)	24.5 ± 9.2	(6, 42.9)	<0.001
Indexed RAV _max_, in ml/m^2^	52.0 ± 12.0	(28, 76)	51.2 ± 10.0	(31.2, 71.2)	0.678	37.6 ± 11.5	(14.6, 60.6)	35.1 ± 9.5	(16, 54.1)	0.181	38.3 ± 10.9	(16.6, 60.1)	35.7 ± 9.7	(16.3, 55)	0.142
Indexed RAV_p−ac_, in ml/m^2^	40.1 ± 10.4	(19.3, 60.9)	36.5 ± 7.1	(22.3, 50.7)	0.021	29.6 ± 9.2	(11.2, 48)	24.9 ± 6.8	(11.3, 38.5)	0.001	30.4 ± 9.4	(11.6, 49.3)	26.1 ± 8.4	(9.3, 43)	0.07
Indexed RAV_min_, in ml/m^2^	26.9 ± 8.9	(9, 44.8)	23.1 ± 5.8	(11.5, 34.8)	0.005	18.7 ± 6.7	(5.3, 32.2)	15.4 ± 4.5	(6.3, 24.5)	0.001	19.6 ± 8.2	(3.2, 36)	16.3 ± 6	(4.3, 28.3)	0.011
Conduit EF, %	23 ± 9	(4, 41)	28 ± 11	(6, 50)	0.003	21 ± 10	(1, 42)	28 ± 11	(6, 50)	<0.001	21 ± 9	(2, 39)	27 ± 10	(6, 48)	<0.001
Booster pump EF, %	33 ± 11	(11, 55)	36 ± 10	(15, 57)	0.092	37 ± 8	(21, 52)	39 ± 9	(22, 57)	0.152	37 ± 11	(15, 58)	37 ± 11	(15, 60)	0.660
Total EF, %	49 ± 10	(29, 68)	54 ± 9	(36, 73)	<0.001	50 ± 9	(32, 68)	56 ± 9	(38, 73)	0.001	49 ± 11	(27, 72)	54 ± 10	(34, 75)	0.010

Lower/upper limits calculated as mean ± 2 SD; RAV_max_, maximal right atrial volume; RAV_p−ac_, right atrial volume before atrial contraction; RAV_min_, minimal right atrial volume; Indexed volumes are calculated by the corresponding volume in mL divided by BSA in m^2^; Conduit EF, Conduit right atrial emptying fraction: 100% × (RAV_max_ − RAV_p−ac_)/RAV_max_; Booster pump EF, Booster pump right atrial emptying fraction: 100% × (RAV_p−ac_ − RAV_min_)/RAV_p−ac_; Total EF, total right atrial emptying fraction: 100% × (RAV_max_ − RAV_min_)/RAV_max_.

### Age Related Changes in LA and RA parameters

Correlations between age and parameters of LA or RA are shown in Table 8. Age was mildly to moderately correlated with the size of the LA and RA (|R| from 0.074 to 0.559). Age also correlated positively with LA volume (R = 0.329, 0.518, 0.259 for LAV_max_, LAV_p−ac_, and LAV_min_, respectively, P < 0.001, <0.001, and =0.003, respectively), while it correlated only mildly with RA maximal volume (R = −0.220, 0.061, −0.092, and P = 0.011, 0.479, and 0.288, respectively). There was a negative correlation between age and atrial conduit EF, and a positive correlation between age and atrial booster pump EFs for both atria (All P < 0.001); however total EFs were not correlated with age (P = 0.568 for LA and P = 0.376 for RA).

**Table 8:** Correlation between age and atrial parameters (all volume parameters were measured by the short axis method) (n = 135).

Parameter	Correlation coefficient	P
LA Long. − 2ch [mm]	0.337	<0.001
LA Trans. − 2ch [mm]	−0.145	0.096
LA Long. − 3ch [mm]	0.559	<0.001
LA AP − 3ch [mm]	0.207	0.016
LA Long. − 4ch [mm]	0.303	<0.001
LA Trans. − 4ch [mm]	0.075	0.385
LAV_max,_ in ml	0.329	<0.001
LAV_p−ac_, in ml	0.518	<0.001
LAV_min_, in ml	0.259	0.003
LA Conduit EF, %	−0.550	<0.001
LA Booster pump EF, %	0.485	<0.001
LA Total EF, %	0.049	0.568
RA Long. − 2ch [mm]	0.282	0.001
RA Trans. − 2ch [mm]	−0.501	<0.001
RA Long. − 4ch [mm]	0.124	0.152
RA Trans. − 4ch [mm]	0.074	0.397
RAV _max_, in ml	−0.220	0.011
RAV_p−ac_, in ml	0.061	0.479
RAV_min_, in ml	−0.092	0.288
RA Conduit EF, %	−0.475	<0.001
RA Booster pump EF, %	0.291	0.001
RA Total EF, %	−0.077	0.376

Long., Longitudinal dimension; Trans, Transverse dimension; AP, Antero-posterior dimension; 2ch, 2-chamber view; 3ch, 3-chamber view; 4ch, 4-chamber LAV_max,_ maximal left atrial volume; LAV_p−ac_, left atrial volume before atrial contraction; LAV_min,_ minimal left atrial volume; LA Conduit EF, Conduit left atrial emptying fraction; LA Booster EF, Booster left atrial emptying fraction; LA Total EF, total left atrial emptying fraction. RAV_max_, maximal right atrial volume; RAV_p−ac_, right atrial volume before atrial contraction; RAV_min_, minimal right atrial volume; RA Conduit EF, Conduit right atrial emptying fraction; RA Booster EF, Booster right atrial emptying fraction; RA Total EF, total right atrial emptying fraction.

### Inter- and Intra-observer Variability

Inter-and intra-observer variability are shown in Tables 9, 10, and 11. Inter- and intra-observer variabilities in atrial dimensional parameters were moderate. Compared with the SAX method, variability was lower by the area-length method in RA 4-ch view, while it was greater by the bi-plane area-length method compared with other measuring methods.

**Table 9:** Inter- and intra-variability of the atrial dimensions (n = 135).

Parameter	Intra-observer consistency (limits of agreement)	CoV	Inter-observer Bias (limits of agreement)	CoV
LA Long. − 2ch [mm]	0.69 (0.41, 0.85)	7.11	0.66 (0.36, 0.84)	8.74
LA Trans. − 2ch [mm]	0.67 (0.40, 0.87)	8.64	0.62 (0.34, 0.82)	11.16
LA Long. − 3ch [mm]	0.91 (0.80, 0.96)	3.63	0.81 (0.6, 0.91)	5.81
LA AP − 3ch [mm]	0.69 (0.41, 0.85)	8.78	0.59 (0.26, 0.80)	10.60
LA Long. − 4ch [mm]	0.83 (0.64, 0.92)	3.82	0.63 (0.32, 0.82)	5.62
LA Trans. − 4ch [mm]	0.84 (0.67, 0.93)	5.64	0.71 (0.44, 0.86)	7.56
RA Long. − 2ch [mm]	0.76 (0.67, 0.86)	10.59	0.66 (0.47, 0.79)	9.52
RA Trans. − 2ch [mm]	0.91 (0.80, 0.96)	18.97	0.86 (0.71, 0.94)	8.74
RA Long. − 4ch [mm]	0.78 (0.56, 0.90)	5.12	0.59 (0.26, 0.80)	7.98
RA Trans. − 4ch [mm]	0.87 (0.72, 0.94)	5.52	0.77 (0.53, 0.89)	7.59

CoV, coefficient of variation. LA, left atrium; RA, right atrium; Long., Longitudinal dimension; Trans, Transverse dimension; AP, Antero-posterior dimension; 2ch, 2-chamber view; 3ch, 3-chamber view; 4ch, 4-chamber view.

**Table 10:** Inter- and intra-variability in left atrial volumes (n = 135)

Parameter	Short axis method				Bi-plane area-length method			
	Intra-observer consistency (limits of agreement)	CoV	Inter-observer Bias (limits of agreement)	CoV	Intra-observer consistency (limits of agreement)	CoV	Inter-observer Bias (limits of agreement)	CoV
LAV_max_, in ml	0.97 (0.93, 0.99)	3.40	0.89 (0.76, 0.95)	5.89	0.77 (0.53, 0.89)	8.84	0.77 (0.54, 0.89)	8.79
LAV_p−ac_, in ml	0.92 (0.83, 0.97)	6.49	0.86 (0.7, 0.94)	9.33	0.87 (0.72, 0.94)	8.41	0.88 (0.75, 0.95)	8.43
LAV_min_, in ml	0.94 (0.87, 0.97)	6.18	0.89 (0.77, 0.95)	8.65	0.93 (0.84, 0.97)	8.68	0.85 (0.67, 0.93)	11.71

LAV_max_, maximal left atrial volume; LAV_p−ac_, left atrial volume before atrial contraction; LAV_min_, minimal left atrial volume.

**Table 11:** Inter- and intra-variability in right atrial volumes (n = 135).

Parameter	short axis method				Bi-plane area-length method				Area-length method (4-chamber)			
	Intra-observer consistency (limits of agreement)	CoV	Inter-observer Bias (limits of agreement)	CoV	Intra-observer consistency (limits of agreement)	CoV	Inter-observer Bias (limits of agreement)	CoV	Intra-observer consistency (limits of agreement)	CoV	Inter-observer Bias (limits of agreement)	CoV
RAV_max_, in ml	0.94 (0.87, 0.98)	6.94	0.93 (0.84, 0.97)	7.50	0.90 (0.78, 0.96)	8.33	0.88 (0.75, 0.95)	8.88	0.95 (0.88, 0.98)	7.88	0.96 (0.91, 0.98)	6.44
RAV_p−ac_, in ml	0.78 (0.56, 0.90)	15.72	0.77 (0.53, 0.9)	17.42	0.85 (0.66, 0.93)	14.68	0.86 (0.69, 0.94)	14.18	0.94 (0.87, 0.97)	9.85	0.92 (0.83, 0.97)	11.11
RAV_min_, in ml	0.86 (0.69, 0.94)	15.18	0.84 (0.67, 0.93)	16.44	0.82 (0.61, 0.93)	20.43	0.76 (0.49, 0.9)	22.76	0.89 (0.76, 0.95)	18.08	0.84 (0.66, 0.93)	21.84

RAV_max,_ maximal right atrial volume; RAV_p−ac_, right atrial volume before atrial contraction; RAV_min,_ minimal right atrial volume.

## Discussion

The present study provided comprehensive reference values for the atrial size and function by SSFP sequence in a population of healthy Chinese volunteers with a wide age range. In addition to providing normal reference standard values, we also found that the left or right atrial volume measured by area-length method was considerably lower than that produced by the SAX volume method, and gender and age have a considerable impact on atrial phasic function, especially on the conduit emptying function and booster pump function.

CMR is an accurate quantitative tool for ventricular and atrial volume and function, based on multi-slice 2D volume acquisition. The SSFP sequence has high signal-to-noise ratio, good myocardium-to-blood pool contrast, and is used routinely in a clinical setting. SSFP at 3.0T further improved the signal-to-noise contrast and could potentially have high spatial resolution to delineate thin-walled chambers. In recent years, new techniques, such as GRE shimming or short TR, have been introduced to increase the robustness of SSFP at 3.0T^28^ and decrease banding artefacts. The normal reference values for atrial size and volume have been investigated at 1.5T in past years in a population of European descent^20, 21, 29^. Only a recent study in a Singaporean Chinese population reported reference values for the left atrium volume, total left atrial ejection fraction, and right atrium area index measured by CMR at 3.0T^30^. To the best of our knowledge, the present study is the first to investigate both left and right atrial volume and phasic function systematically.

Reference LA dimensions and volume have been studied in normal populations before. However, data derived from earlier sequences, such as TSE or GRE, are not truly comparable to SSFP sequences. Also, data acquired by SSFP sequence with prospective ECG gating not fully covering diastole are not comparable with retrospective ECG gating, which is the current routine in clinical practice^31, 32^. Therefore, very limited LA data could be comparable to our present study. We found the LA dimensions to be similar to those reported by Maceira, *et al*.^20^; e.g., the upper limit for the LA antero-posterior dimension in the Chinese population was 41 mm, comparable to the 42 mm for those of European descent. The LA absolute maximal volume in our study was lower than that reported for people of European descent, but was accounted for by the BSA. However, the LA maximal volume in our study was lower than that in the Singaporean Chinese population, even after adjusting by BSA (LA maximal volume index: 50 ± 10 mL/m^2^ *vs*. 38.2 ± 10.1 mL/m^2^)^30^. The reason for this difference is unknown, as the sequence parameters, analysis methods used, and ethnicities of the study population are similar. Left atrial phasic function is a very interesting topic in cardiovascular disease^10, 24, 33^. However, few previous studies showed normal references in healthy populations. The total LAEF in our study was similar to the data presented in the study by Marceira *et al*. (60 ± 8% *vs*. 59 ± 8%)^20^. In addition, our study demonstrated gender specific LA phasic function systemically, which was not fully explored in previous studies.

Few previous studies investigated RA size and volume. Accurate RA volume is difficult to estimate by 1D or 2D measurements. RA size, as measured by volume, was greater in males than in females, despite similar single dimension measurements in our study. Dimensions derived from the 4-ch view or RV 2-ch view were generally lower than those in previous data and the indexed dimensions were slightly higher than the indexed dimensions in people of European descent^21^. In our study, absolute RA maximum volumes measured on short axis slices were lower than those measured in people of European descent; however, the indexed values were similar (51.6 ± 11.0 mL/m^2^; versus Sievers’s 52.8 ± 16.3 mL/m^2^, and Maceira’s 54 ± 10.3 mL/m^2^)^20, 29^. This was the first study to demonstrate the phasic function of RA in a normal population. A recent study showed that the RA emptying fraction was an independent and robust indicator for mortality in patients with pulmonary hypertension^16^. This study suggested the potential importance of RA phasic function evaluation in future studies.

While SAX method is considered the gold standard for measuring atrial volume without geometric assumption, the area-length method is a simple alternative. Previous comparisons of these two methods based on small normal populations showed good correlation with the LA volume^34^. Our study validated the area-length method further in a Chinese population and demonstrated that the area-length method gives a reasonable estimation of LA volume, although the absolute volume is lower than the true volume, as measured by the SAX method. Left atrial conduit function estimated by the SAX method was significantly higher than that estimated by the area-length method. Therefore, the LA volume and function derived by the area-length method should be interpreted cautiously, especially when these parameters are the main indications for the CMR examination in patients with cardiac remodelling^23^. In contrast to the LA, area-length methods for estimating the RA volume have not been studied in depth. In our study, neither the area-length of the 4-ch nor the bi-plane area-length from 4-ch and RV 2-ch could estimate the RA volume accurately. The RA volume was underestimated remarkably by the area-length method comparing with the SAX method. Thus, if accurate RA volume measurement is necessary, the SAX method is preferred.

Generally, the absolute LA dimensions and volume were greater in males than in females; however, indexing by BSA reduced the differences. The LA maximal volume index was actually larger in females than in males. Our results were in accordance with previous studies^20, 21, 29, 30, 32, 35^. Right atrial size and volume were greater in males than in females, and these differences also decreased after indexing by BSA, except the RA minimal volume and RA pre-contraction volume. Phasic function was also associated with gender. Males had lower conduit empty fractions (EFs) for both atria. This phenomenon could indicate a gender difference in diastolic ventricular function. The genders have different blood pressures; therefore, whether the conduit empty function is associated with blood pressure or just gender requires further study.

The age related changes in atrial size and function were inconsistent with previous reports. In Sievers’s study^32^, LAV_max_ did not correlate with age in healthy volunteers. Maceria *et al*.^20^ also did not find an association between the LA volume with age, and age was not an independent indicator for the LA volume. However, the LA transverse and long dimensions were related with age. In a recently reported Singaporean Chinese population, the LA maximal volume did not correlate with age in either gender^30^. Meanwhile, another study in a large European population, including a younger age range, demonstrated a significant correlation between age and LA maximum volume^35^. The differences among these studies might be caused by a bias in population selection, inconsistent analysis methods for the LA volume (most of these were derived by the area-length method), and limited sample size with different age groups. In our study, we correlated age with LA volume by the SAX method and our population covered a wide age range. Our study confirmed there was a moderate positive correlation between age and the LA maximal volume or LA pre-contraction volume, while the correlation between age and RA maximal volume was only slightly negative. Interestingly, we found a very significant correlation between age and the LA phasic function, which was not observed in previous studies. Both left and right atrial conduit EFs correlated negatively with age, whereas booster pump EFs correlated positively with age. These data demonstrated the impact of age on ventricular diastolic function and atrial remodelling with aging.

In summary, in the present study, we investigated the reference values of the left and right atrial dimension, volume, and phasic function using the state of art SSFP sequence at 3.0T MRI in a healthy Chinese population. The SAX method provided more accurate values for the atrial volume and showed better reproducibility compared with the area-length method, especially for the right atrial volume. Therefore, additional short axis slices for the atrium are necessary if the atrial volume is the main question in a clinical study. Indexing to BSA is important to account for certain gender differences. We also demonstrated that age is related to atrial geometry and atrial phasic function. These findings emphasized the potential utility of evaluating atrial phasic function in future studies.
